# Genetic Characterization of *Toxoplasma gondii* from Wild Rodents in Sichuan Province, Southwestern China

**Published:** 2019

**Authors:** XinLei WANG, Ling DONG, Li ZHANG, Yan LV, Qian LI, HaiLong LI

**Affiliations:** 1. College of Basic Medicine, Dali University, Dali, China; 2. Jinci College of Shanxi Medical University, Taiyuan, China

**Keywords:** *Toxoplasma gondii*, Genetic typing, PCR-RFLP, Wild rodents, China

## Abstract

**Background:**

Wild rodents are the intermediate hosts of *Toxoplasma gondii*. The distribution of genetic diversity of *T. gondii* in wild rodents is of importance to understand the transmission of this parasite. This study aimed to genetically characterize *T. gondii* isolates from wild rodents in Sichuan province, southwestern China in 2013.

**Methods:**

Genomic DNA was extracted from 10 g wild rodents’ brain samples. Semi-nested PCR and multilocous PCR-RFLP technology were performed to examine genetic diversity of *T. gondii* isolates as described previously.

**Results:**

Overall, 181 brain tissues of different wild rodents, including *Eothenomys miletus* (n=88), *Crocidura attenuate* (n=9), *Rattus rattus sladeni* (n=46), *Mus musculus Linnaeus* (n=6) and *R. niviventer* (n=32) were tested for *T. gondii* DNA, respectively. Six of them were positive for the *T. gondii* B1 gene by semi-nested PCR amplification, 4 showed complete genotyping results for all 11 polymorphic loci (SAG1, SAG2, alt. SAG2, SAG3, BTUB, GRA6, L358, PK1, C22-8, C29-2 and Apico) by PCR-RFLP, determined to represent a potential new genotype (http://toxodb.org/toxo/).

**Conclusion:**

These results documented genetic characterization of *T. gondii* in wild rodents from Sichuan province, and enriched the genetic diversity of *T. gondii* in China.

## Introduction

*Toxoplasma gondii* is an obligatory intracellular protozoan that causes a widespread zoonosis-toxoplasmosis (1). This disease is mostly subclinical and unnoticed in healthy individuals, however, other immunocompromised patients such as AIDS patients or cancer patients with undergoing immuno-suppressive therapy can get severe diseases, even death. *T. gondii* can infect the fetus via the placenta by transplacental transmission and damage the baby.

In general, clinical presentations of toxoplasmosis are associated with the *T. gondii* genotypes. Four major clonal lineages types (I, II, III and 12) of *T. gondii* were classified by genetic polymorphism (2–4). However, in China, the Chinese I (Toxo DB9#) is the main genotype (5–8).

Wild rodents are the intermediate hosts of *T. gondii* and play important role in the transmission. There were different *T. gondii* genotypes in distinct areas of wild rodents in China (9–11). Sichuan with a unique ecosystem is one of provinces located in southwestern China. However, there is little genetic information on *T. gondii* diversity in this province. This research focused on the genetic characterization of *T. gondii* isolates from wild rodents in Sichuan Province. These findings would provide baseline data for improving prevention and control of *T. gondii* infection in wild rodents.

## Materials and Methods

Wild rodents were collected from four villages, Liangshan Yi Autonomous Prefecture of Sichuan Province, southwestern China in 2013: Binggu town (26°46′09.46″N, 102°5′14.57″E, 1142 m), Malong village (26°55′35.90″N, 101°5′58.63″E, 1992m), Pingchuan Nuomigou village (27°37′38.77″N, 101°48′10.04″E, 2321 m) and Boke Luona village (28°09′57.50″N, 100°55′16.84″E, 2750 m) (Table 1).

**Table 1:** The prevalence of *Toxoplasma gondii* infection from wild rodents in Sichuan Province, Southwestern China

***Variable***	***No. Positive***	***No. examined***	***Prevalence (%)***
Species			
*Eothenomys miletus*	1	88	1.14
*Crocidura attenuate*	1	9	11.11
*Rattus rattus Sladeni*	2	46	4.35
*Mus musculus Linnaeus*	1	6	16.67
*Rattus niviventer*	1	32	3.13
Region			
Miyi District	2	26	7.69
Binggu District	2	25	8.00
Pingchuan District	2	54	3.70
Boke area	0	76	0.00
Age			
Juvenile group	0	2	0.00
Sub-adults group	0	4	0.00
Adults group	6	175	3.43
Total	6	181	3.31

These four locations were located in the southwest of Sichuan Province, sharing borders with Yunnan Provinces in the south, with an average annual rainfall of 1000~1100 mm.

Overall, 181 wild rodents were captured including *Eothenomys miletus* (n=88), *Crocidura attenuate* (n=9), *Rattus rattus sladeni* (n=46), *Mus musculus Linnaeus* (n=6) and *R. niviventer* (n=32). These rodents were divided into three age groups according to their ages: juvenile group (with the body length ≤110 mm), sub-adult group (with the body length of 111–150 mm), and adult group (with the body length >115 mm) (10).

All rodents were handled in accordance with regulations laws required by the Animal Ethics Procedures and Guidelines of the People’s Republic of China. This study received ethical approval by the Animal Ethics Committee of Dali University.

Ten grams of each animal’s brain tissue was digested with proteinase K for 2 h, then TIANamp Genomic DNA kit (Tiangen ^™^, Beijing, China) was used to extract the genomic DNA. Semi-nested PCR targeting the *T. gondii* B1 gene was performed to detect *T. gondii* infection as described previously (10). DNA samples showing positive *T. gondii* B1 gene amplification were then used for further genetic characterization.

Multilocus PCR-RFLP method targeting the 11 genetic markers (i.e., SAG1, 5′-and 3′-SAG2, alternative SAG2, SAG3, BTUB, GRA6, c22-8, c29-2, L358, PK1, and Apico) was used to genetically characterize the positive DNA samples as described previously (10, 12–14).

### Statistical analysis

The prevalence of *T. gondii*-infected wild rodents among different variables including species, region and age were analyzed using multi-nomial logistic regression by SPSS for Windows (Release 16.0 standard version, Chicago, IL, USA). Statistical differences were considered to show statistically significant when *P*< 0.05.

## Results

The prevalence of *T. gondii* B1 gene in wild rodents as determined by PCR amplification is shown in Table 1. Six out of 181 (3.31%) DNA samples were positive for the *T. gondii* B1 gene. The four rodent species, namely *E. miletus*, *C. attenuate*, *M. musculus Linnaeus* and *R. niviventer* showed 1 positive sample, respectively, while *R. rattus sladeni* had 2 positive samples. The 6 positive samples were distributed in Miyi, Binggu and Pingchuan district. However, there was no positive sample found in Boke. All the positive rodents belong to adults group. There was no statistical difference in *T. gondii* prevalence among species, regions and age.

Among the 6 *T. gondii*-positive DNA samples, only 4 positive DNA samples were genotyped completely at 11 genetic loci. The 4 samples represent a new genotype (Table 2). Due to low DNA concentration, other 2 samples were identified at less than 6 loci, and the results were not shown.

**Table 2:** Summary of genotyping of *Toxoplasma gondii* in wild rodents from Sichuan province in China

***Isolate ID***	***Host***	***Tissue***	***Location***	***SAG1***	***5’+3’SAG2***	***Altern ative SAG2***	***SAG3***	***BTUB***	***GRA6***	***c22-8***	***c29-2***	***L358***	***PK1***	***Ap ico***	***Genotype***
GT1	Goat		USA	I	I	I	I	I	I	I	I	I	I	I	Reference,ToxoDB#10
PTG	Sheep		USA	II/III	II	II	II	II	II	II	II	II	II	II	Reference, ToxoDB#1
CTG	Cat		USA	II/III	III	III	III	III	III	III	III	III	III	III	Reference, ToxoDB#2
MAS	Human		France	u-1	I	II	III	III	III	u-1	I	I	III	I	Reference,ToxoDB#17
TgCgCa1	Cougar		Canada	I	II	II	III	II	II	II	u-1	I	u-2	I	Reference,ToxoDB#66
TgCatBr5	Cat		Brazil	I	III	III	III	III	III	I	I	I	u-1	I	Reference,ToxoDB#19
TgWtdSc40	Deer		USA	u-1	II	II	II	II	II	II	II	I	II	I	Reference, ToxoDB#5
TgCatBr64	Cat		Brazil	I	I	u-1	III	III	III	u-1	I	III	III	I	Reference,ToxoDB#11
TgRsCr1	Toucan		CostaRica	u-1	I	II	III	I	III	u-2	I	I	III	I	Reference, ToxoDB#52
TgSc9	Rattus rattus slade	Brain	Binggu	I	I	I	II	I	I	II	I	I	I	I	New genotype
TgSc12	Rattus rattus slade	Brain	Binggu	I	I	I	II	I	I	II	I	I	I	I	New genotype
TgSc49	Eothenomysmiletus	Brain	Miyi	I	I	I	II	I	I	II	I	I	I	I	New genotype
TgSc74	Crocidura attenuata	Brain	Miyi	I	I	I	II	I	I	II	I	I	I	I	New genotype

## Discussion

*T. gondii* is widely distributed throughout the world and has rich genetic diversity, due to the factors such as hosts and geographical distribution as well as the population structure. In Sichuan Province, seroprevalence of *T. gondii* infection in yaks (*Bos grunniens*) was 25.5% (54/212) and 33.7% (85/252) in 2012 and 2013, respectively (15); in household dogs the prevalence was 3.5% (11/34) in 2012 (16). Approximately 200 *T. gondii* genotypes have been identified and the four major genotypes in the world were ToxoDB #1, ToxoDB #2, ToxoDB #3 and ToxoDB #10 (17). The *T. gondii* genotypes prevailing in China are ToxoDB #1, ToxoDB #2, ToxoDB #3, ToxoDB #9, ToxoDB #10, ToxoDB #20, ToxoDB #204, ToxoDB #205 and ToxoDB #225 (7,18–20). ToxoDB #9 was identified in pigs in Sichuan Province (14).

The study area in the present study is a region of Sichuan Province with plain, tableland, high hills, lower and medium height mountains that has typical complex and diverse landscape and rich in biodiversity. However, little information about the prevalence and genetic characterization of *T. gondii* is known in this ecological diversity environment. Rat is one of the food sources of cats, and naturally infected rat plays an important role in *T. gondii* transmission. The seroprevalence of *T. gondii* in rats in southern China was 3.2% (7/217) by modified agglutination test (MAT) (13); in eastern China, *T. gondii* prevalence in rats was 23.6% (29/123) by B1 gene-targeted PCR amplification, and 7 PCR-positive samples were completely genotyped and they were identified as genotype China 1 (ToxoDB# 9)(10). In another study, 11 out of 183 were found to be positive for the *T. gondii* B1 gene in wild rodents from northwest China, 4 samples belonged to ToxoDB Genotype #10 and two samples were identified as two new genotypes (11). Our findings indicated that 6 out of 181 wild rodents were positive for the *T. gondii* B1 gene and all the 4 successfully genotyped belong to the same type, representing a potentially new genotype. This is the first report of genetic typing of *T. gondii* isolates in rats in Sichuan Province.

## Conclusion

The prevalence of *T. gondii* in rats was 3.31% in Sichuan Province. All the four *T. gondii* DNA samples determined to represent a potentially new genotype, and it is the first report about the genetic characterization of *T. gondii* of rats in Sichuan Province China. These findings will provide reference for further studies of the genetic diversity of *T. gondii* in China.
